# The stigma towards mental illness: Portuguese validation of the Opening Minds Stigma Scale for Healthcare Providers (OMS-HC)

**DOI:** 10.3389/fpsyg.2024.1359483

**Published:** 2024-03-07

**Authors:** Maria Beatriz P. Moreira, Helena P. Pereira, Inês N. Torres, Sílvia Marina, Miguel Ricou

**Affiliations:** ^1^Faculty of Medicine, University of Porto, Porto, Portugal; ^2^Center for Health Technology and Services Research, Faculty of Medicine, University of Porto, Porto, Portugal

**Keywords:** mental-health-related stigma, measurement instruments, psychometrics, mental illness, cross-sectional study

## Abstract

**Background:**

Stigma toward mental illness significantly contributes to a lower quality of healthcare that can be provided. There are few studies on this topic in Portugal, so validating a scale that can evaluate and study the stigma is paramount. The aim of this study was to validate the Opening Minds Stigma Scale for Portuguese healthcare professionals.

**Methods:**

A total of 503 participants were included in this study, and the majority was female (81.1%). The sample consisted mainly of psychologists (39.4%) and physicians (30.8%). Reliability and validity analyses were conducted and included exploratory factor analysis (EFA) and confirmatory factor analysis (CFA).

**Results:**

Our results suggest that a 12-item model was the most appropriate (RMSEA = 0.026, SRMR = 0.057, CFI = 0.979, TLI = 0.973, GFI = 0.955) compared to our 15-item model and the original model. Items 8, 9 and 10 were removed. The 12-item scale’s internal consistency was adequate (α = 0.71; ω = 0.72).

**Conclusion:**

The 12-item model of the scale showed good reliability and validity and is appropriate for use with Portuguese healthcare professionals.

## Introduction

Stigma is a complex concept involving labeling, stereotyping, separation, status loss, and discrimination toward people defined as “other” (Link and Phelan, 2001). Stigmatizing acts can be either conscious or unconscious and can be committed against a person categorized as “other” based on gender, race, sexuality, disability, condition or any other criteria that can separate groups of people. Alternatively, individuals can stigmatize themselves (Link and Phelan, 2001). Stigma can significantly impact a person’s well-being and ability to meet their basic needs.

A group that is frequently stigmatized is individuals with mental illness (Corrigan et al., 2014). Society often otherizes them, perpetuating stereotypes that label them “dangerous” or “weird.” Consequently, they may be excluded from communities, finding it more challenging to obtain employment, and be treated as “less than” and less capable of being productive members of society (Rössler, 2016). As a result, many people with mental illness are pushed into a lower socio-economic status, exacerbating their struggles. Symptoms of mental illness are frequently considered shameful, dangerous, faked and incurable, while the person suffering from them is often labeled as unstable, irrational, lazy and helpless (Fabrega, 1990). The root of this issue stems from history, where mental illness was often attributed to possession by spirits or demons, sinfulness or labeling individuals as witches (Fabrega, 1990).

Educating the general public and promoting of contact with people with mental illness is crucial in addressing and reducing stigmatization (Corrigan et al., 2012). Such education may change the perspectives of people on certain issues, but it is more challenging to change their attitudes (Hagerty et al., 2005). Likewise, although healthcare education has the potential to increase knowledge about mental illness, personal biases can be influenced by society and require significant effort to be changed (Petkari, 2017). So, healthcare professionals are not immune to stigma and may exhibit it in their interactions with individuals with mental illness (Schulze, 2007). This stigma can significantly impact the access of patients to healthcare and can also delay the detection of problems and necessary interventions, exacerbating the health risks associated with low socio-economic status (Schulze, 2007; Corrigan et al., 2014). So, addressing stigma in healthcare professionals is also critical in minimizing barriers to accessing healthcare and enhancing the overall quality of services provided.

The professionals often believe that they do not have stigmatizing attitudes. Therefore, addressing stigma requires an increase in awareness of the problem, both at a social and individual level (Munisami et al., 2021). Likewise, research is needed to study the extent to which stigma against mental illness is prevalent among healthcare providers. So, considering this need the Opening Minds Stigma Scale for Healthcare Providers (OMS-HC) was the first scale developed to assess stigma toward mental illness specifically among healthcare professionals (Kassam et al., 2012). A team of researchers from the University of Calgary in Canada developed this scale, and, since then it has been validated in several countries, including Malaysia (Fernandez et al., 2016), Singapore (Chang et al., 2017), Italy (Destrebecq et al., 2018), Pakistan (Laraib et al., 2018), Bahrain (Al Saif et al., 2019), Chile (Sapag et al., 2019), Hungary (Őri et al., 2020), Germany (Zuaboni et al., 2021), Iran (Movahedi et al., 2022) and Brazil (Carrara et al., 2022, 2023).

The original study of the scale showed satisfactory internal consistency for the total scale (α = 0.79) and lower internal consistency for the dimensions proposed for it (α < 0.70 for all extracted factors; Modgill et al., 2014).

The OMS-HC validation studies conducted in European countries (Germany, Hungary and Italy) obtained slightly different results (Destrebecq et al., 2018; Őri et al., 2020; Zuaboni et al., 2021). The German study showed good internal consistency for the total scale (α = 0.74) and lower for the three factors (α < 0.70), and the authors maintained the original factor structure (Zuaboni et al., 2021). The Hungarian study (Őri et al., 2020) also had good internal consistency (α = 0.73) and lower internal consistency for the three factors of the factor structure (α < 0.70). However, its results showed that one of its items should be removed (Őri et al., 2020). In addition, although they obtained good results for a three-factor structure, their results indicated good results for a bifactor structure with one global factor and three specific factors (Őri et al., 2020). The Italian study (Destrebecq et al., 2018) tested a 15-item version with a three-factor structure and a 12-item structure with two factors. In this study, both the three factors and the two factors showed good internal consistency (alpha ranging from 0.74 to 0.86).

The validation study for the Brazilian Portuguese version (Carrara et al., 2023) of the scale revealed good internal consistency of the total scale (α = 0.74). The results of this study indicated that the four-factor structure would be the most suitable for this population, and they also tested a five-factor and single-factor structure (Carrara et al., 2023). Only one of the factors extracted in this study had a good Cronbach’s alpha (α >0.70; Carrara et al., 2023).

Also, the factor structure of the OMS-HC was also tested in 32 European countries, including a sample of Portuguese psychiatrists (Őri et al., 2023). The bifactor structure was considered more suitable for most countries in comparison to the original structure based on a correlated model. This reveals the need for further research in this field, namely with Portuguese healthcare professionals. Thus, this study aims to validate the OMS-HC scale in order to use it to analyze the prevalence of stigma toward mental health among healthcare professionals in Portugal.

## Methods

### Design and sample

This study is cross-sectional and observational, with the aim of validating a scale for healthcare professionals in Portugal. The primary variable being measured in this scale is the stigma toward mental illness among healthcare professionals. Participants for this study were recruited through a snowball method. Healthcare professionals were invited to participate in the study through a link shared on online platforms. Participants themselves gave the link to new referrals, thus inviting them to participate in the study.

### Instruments

The Opening Minds Stigma Scale for Healthcare Providers (OMS-HC) consists of 15 items and accurately measures the presence of stigma (Modgill et al., 2014). The scale is divided into three subscales: “Attitudes of health care providers toward people with mental illness” (comprising the items 1, 9, 10, 11, 13 and 15), “Attitudes of healthcare providers toward disclosure and help-seeking” (comprising the items 3, 4, 5 and 8) and “Attitudes of healthcare providers toward social distance” (comprising the items 2, 6, 7, 12 and 14). This is a Likert Scale with five response options: “Strongly Disagree,” “Disagree,” “Neither Agree or Disagree,” “Agree” and “Strongly Agree.” Items 2, 6, 7, 8 and 14 require reverse scoring. After completing the questionnaire, each participant had a score ranging from 15 to 75, with the lower scores indicating less stigma. The scale’s Cronbach’s alpha is 0.79, while the Cronbach’s alphas of the subscales are 0.68, 0.67 and 0.68, respectively (Modgill et al., 2014).

The 20-item Social Desirability Scale [Escala de Desejabilidade Social (EDS-20)] was used to determine whether participants were answering based on social desirability rather than their real opinions (Almiro et al., 2017). This Portuguese scale combines features of the Marlowe-Crowne scale and the Social Desirability/Lie subscale of the Eysenck Personality Questionnaire. It is a dichotomous scale with 20 items, where participants answer “Yes” or “No,” which are scored 0 or 1, respectively. Item 4 is an exception since its score is reversed. The scale yields a score ranging from 0 to 20. Scores above 13 indicate that a person is answering based on social desirability, while scores between 5 and 13 are considered typical and scores below 5 suggest high sincerity. The scales Cronbach’s alpha is 0.82, indicating a good reliability (Almiro et al., 2017).

A socio-demographic questionnaire was developed to collect information on gender, age, education level, profession, professional specialty, years of experience and affiliation with professional associations. It should be noted that the classifications of Portuguese professional associations were considered when identifying professional specialties.

### Procedures

The first step in the study was a cross-cultural adaptation to the Portuguese validation. The original authors were contacted for permission to proceed, and the scale was then translated to European Portuguese. Two individuals external to the study, fluent in Portuguese and English completed the initial translations. The research team merged and reviewed the two translated versions for clarity and consistency. Changes were made to ensure the best possible interpretation of the items. The Portuguese version was back-translated by a native English speaker that was fluent in Portuguese. A comparison between the original and back-translated versions showed minor differences that did not significantly affect the interpretation of the items.

Later, a pre-test was conducted with 17 participants from health-related fields. They were required to complete the questionnaire and provide feedback on the grammatical correctness and understandability of the questions, as well as any other general opinions. Based on the feedback received, changes were made to items 5, 6 and 12 to improve the accuracy of the translation. Pronouns and suffixes were added to items 2, 4, and 7 to enhance inclusivity. Likewise, any formatting or technical difficulties reported by participants were addressed.

A test–retest was conducted with 31 participants who were inquired to provide a code to identify their questionnaire, without compromising anonymity. Participants were then requested to response the questionnaire twice, at a one-week interval, to assess the temporal reliability of the scale.

In the third phase, the questionnaire was available via a link shared in online platforms (e.g., Facebook, Instagram, LinkedIn and Twitter). The questions were answered online through the Google Forms platform. This procedure was selected to increase participation of the healthcare professionals. Data collection was performed from January to February 2023. All questions were mandatory to avoid missing data.

This study was approved by the ethics committee (Comissão de Ética Centro Hospitalar S. João/ Faculdade de Medicina da Universidade do Porto) under number 07/2023. All procedures were conducted following the 1964 Helsinki Declaration, its later amendments, comparable ethical standards and the General Data Protection Regulation (European Commission, 2016). Informed consent was obtained from all participants included and was incorporated into the link for participation in the study. Participation in the study was anonymous and no electronic authentication was required.

### Data analysis

The statistical analysis of the quantitative variables was conducted using the Statistical Package for the Social Sciences software for Windows (IBM SPSS, v. 27.0, Armonk, NY, United States), except for the Confirmatory Factor Analysis, which was performed using JASP v.0.17.1.0.

The study included a characterization of the participants and the scale. The reliability analysis included internal consistency and test–retest analyses. The Validity analysis, specifically the construct validity, consisted both of exploratory factor analysis and confirmatory factor analysis.

Descriptive statistics were calculated for the items of the scale using mean and standard deviation values. Parametric tests were used because a normal distribution of results could be assumed, considering the values of skewness and kurtosis (|Sk| < 3 and |Ku| < 10; Kline, 2015). Pearson’s correlation coefficient was used to examine correlations, with a significance level of 0.05 (Pearson’s *r* = 0.00–0.19 very weak, 0.20–0.39 weak, 0.40–0.59 moderate, 0.60–0.79 strong, 0.80–1.00 very strong).

To analyze the structure of the scale an exploratory factor analysis (EFA) were conducted. Before the EFA, the Kaiser-Meyer-Olkin (KMO) measure and Bartlett’s test of sphericity were calculated to ensure the adequacy of the data for EFA. The KMO was used to assess the adequacy of the matrices for analysis (KMO > 0.60), and Bartlett’s Test of Sphericity was used to ensure that the correlation matrix was not random. The maximum likelihood extraction method and Promax oblique rotation were used to determine the factor structure of the scale (Marôco, 2021b). Factors were extracted using the Kaiser criterion (eigenvalues greater than one) and the scree plot information.

Confirmatory factor analysis (CFA) was conducted to test the models using the maximum likelihood estimation method. Several models were tested to ensure the sensitivity of the analysis with a minimum of 10 observations per item respected (Marôco, 2021a). The adjustment indexes used to evaluate the adequacy of the models included the ratio between chi-squared and degrees of freedom (x^2^/df), with acceptable results ranging between 2.0 and 5.0. The Comparative Fit Index (CFI), Tucker-Lewis Index (TLI) and Goodness-of-Fit Index (GFI) were also used with values ranging from 0 to 1, and values greater than 0.9 suggesting that the model is adequate for the analyzed data. The Standardized Root-Mean-Square Residual (SRMR) and Root-Mean-Square Error of Approximation (RMSEA) were also used, with results below 0.08 indicating acceptable adequacy. The Expected Cross-Validation Index (ECVI) a variant of the Browne-Cudeck criterion (BCC) of a single sample of cross-validation was used (Browne and Cudeck, 1989), as well as the Akaike information criterion (AIC), which indicates the simplicity of the model tested. To improve the adjustment of the models, residual errors were corrected based on the assumptions established by Marôco (2021a).

The internal consistency of the OMS-HC was assessed using Cronbach’s alpha and McDonald’s omega coefficients. Pearson’s correlations among items and among the items and the total scale were also analyzed.

In the test–retest analysis, an Intra-Class Analysis (ICC) between two moments of the scale application were performed. ICC estimates and their 95% confidence intervals were calculated to test the consistency within the scale, with ICC values below 0.50 indicating poor consistency, 0.50–0.75 indicating moderate consistency, 0.75–0.90 indicating good consistency and values above 0.90 indicating excellent consistency. Also, a Pearson’s correlation between the two moments of the scale application and a paired samples t-test were performed (considered a value of *p* < 0.05 to be significant).

## Results

### Sample characteristics

Of the initial sample of 557 responses, 503 were considered. Fifty-four participants were excluded because they did not fulfill one of the inclusion criteria, as they were not healthcare professionals. The samples were randomly divided for exploratory factor analysis and confirmatory factor analysis, as a recommended good practice (Kline, 2015; Marôco, 2021a). For the EFA a sample of 250 were used and a sample of 253 were used in the CFA.

Of the 503 participants, 408 identified as female (81.1%), 91 as male (18.1%), 1 as non-binary (0.2%) and 3 did not respond (0.6%). The mean age of participants was 38.95 (SD = 11.75) years, with the minimum being 22 and the maximum being 75.

The sample consisted of 39.4% psychologists, 30.8% medical doctors, 9.1% nurses, 8.9% dentists, 2.2% pharmacists, 1.4% nutritionists and 8% other healthcare professionals. The mean years of experience were 18.27 (SD = 6.75), and 89.7% of participants were affiliated with their respective professional associations, while 10.3% either had no affiliation or was not applicable. Around 49.7% had a specialty, namely in medicine, psychology or nursing. For the professional group of medical doctors, 36% had a specialty in general and family medicine, 9.6% in psychiatry, 13.7% in internal medicine, 16.4% in surgery and 23.3% in other specialties (e.g., cardiology, dermatology, gynecology and obstetrics, neurology). All the psychologists who indicated their specialty were clinical and health psychologists. In the group of nurses, 22.2% were mental health nurses, while the others (77.7%) were nurses from other specialties, such as rehabilitation, medical-surgical or community nursing.

### Scale structure

Considering the suitability of the data for EFA, Kaiser-Meyer-Olkin (KMO) value was 0.802 and Bartlett’s test of sphericity showed a chi-square of 821.501 and a value of *p* <0.001, indicating that it would be possible to proceed to the EFA.

**Figure 1:**
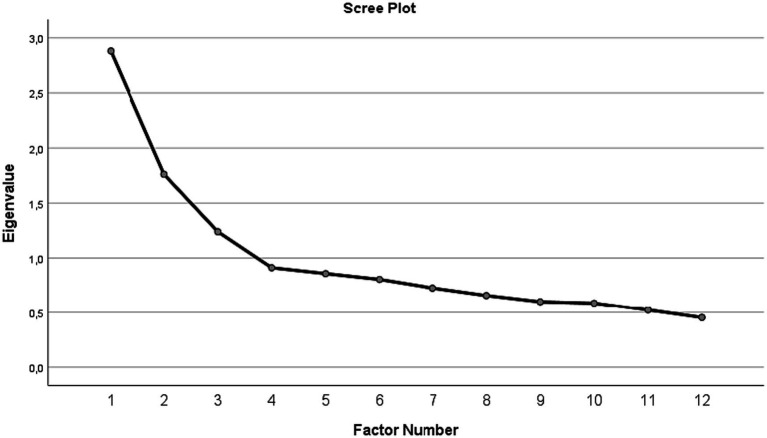
Scree plot showing eigenvalues for each factor, in factor extraction of the 12-item OMS-HC scale version. *N* = 250.

A total of three factors were extracted on the EFA, considering the Kaiser criterion and the scree plot (see Figure 1). Factor one included items 1, 3, 4 and 5, Factor two included items 11, 12, 13 and 15 and Factor three had items 2, 6, 7 and 14. Three of the 15 item, namely, items 8,9, and 10, did not have factor loadings higher than 0.3 on any factor. Items 9 and 10 had very low factor loadings for factor two (0.101 and 0.096, respectively) and item 8 had very low factor loadings for factor three (0.043). These results suggested that a structure consisting of 12 items might be more appropriate. The factor loadings obtained for each factor in 12-item version are shown in Table 1. The 12-item version explained 41.627% of the variance of the items, with factor one explaining 22.931%, factor two explaining 10.082% and factor three explaining 8.614% of the variance, respectively (see Table 1).

**Table 1:** Factor structure of the 12-item OMS-HC scale version.

	Factor 1	Factor 2	Factor 3	*h2*
Item 1	0.398			0.300
Item 3	0.459			0.414
Item 4	0.708			0.512
Item 5	0.730			0.537
Item 2		0.500		0.451
Item 6		0.491		0.374
Item 7		0.715	0.305	0.515
Item 14		0.441	0.233	0.413
Item 11		0.321	0.562	0.422
Item 12		0.314	0.589	0.465
Item 13			0.504	0.377
Item 15			0.495	0.349
Factor Variance	22,931%	10,082%	8,614%	
Eigenvalues	2.881	1.761	1.239	

*N* = 250. Factor loadings below 0.3 are omitted. h2 = Communality values.

### Confirmatory factor analysis

In the confirmatory factor analysis (CFA), we tested three models for the structure of the scale, namely the original structure proposed by the authors, the 15-item EFA structure and the 12-item EFA structure.

We tested the model proposed by the original authors with factor one including items 3,4,5 and 8, factor two including items 1, 9, 10, 11, 13 and 15 and factor three including items 2, 6, 7, 12 and 14. This model did not obtain good adjustment index values. The modification index indicated the need to correlate the residuals of items 13 and 15, and also items 1 and 10. However, the results were still not good, with a chi-squared of 165.005 (df = 85) and a *p* value <0.001, CFI, TLI and GFI values below 0.90, SRMR and RMSEA above 0.08, as can be seen in Table 2.

**Table 2:** Comparison of the results of each model tested in the confirmatory factor analysis.

	x^2^(df)	x^2/^df	*p*	RMSEA	SRMR	CFI	TLI	GFI	AIC	ECVI
Original model	175.005 (81)	2.04	<0.001	0.081	0.093	0.848	0.806	0.895	8296.472	1.158
15-item EFA model	153.095 (86)	1.78	<0.001	0.072	0.078	0.883	0.857	0.909	8252.562	1.089
12-item EFA model	57.969 (51)	1.14	0.234	0.026	0.057	0.979	0.973	0.955	6715.744	0.522

*N* = 253. χ^2^ = Chi-square; χ^2^/df = Chi-square/Freedom degree; RMSEA, Root Mean Square Error of Approximation; SRMR, Standardized Root-Mean-Square Residual; CFI, Comparative Fit Index; TLI, Tucker-Lewis Index; GFI, Goodness-of-Fit Index; AIC, Akaike Information Criterion; ECVI, Expected Cross-Validation Index.

The 15-item model extracted from the EFA with a different factor structure to the original was also tested. This model also did not obtain a good adjustment index values, with CFI and TLI values below 0.90, and RMSEA values above 0.08. To improve model fit values, the modification index showed the need to correlate the residuals of items 13 and 15, items 1 and 10, items 3 and 8, and also items 9 and 2. Only items 13 and 15 belonged to the same factor, which could emphasize the problems with items 8, 9 and 10 in the EFA. With the correlation of residual errors, the model improved in RMSEA values, although the CFI and TLI values were still lower than expected (see Table 2).

**Figure 2:**
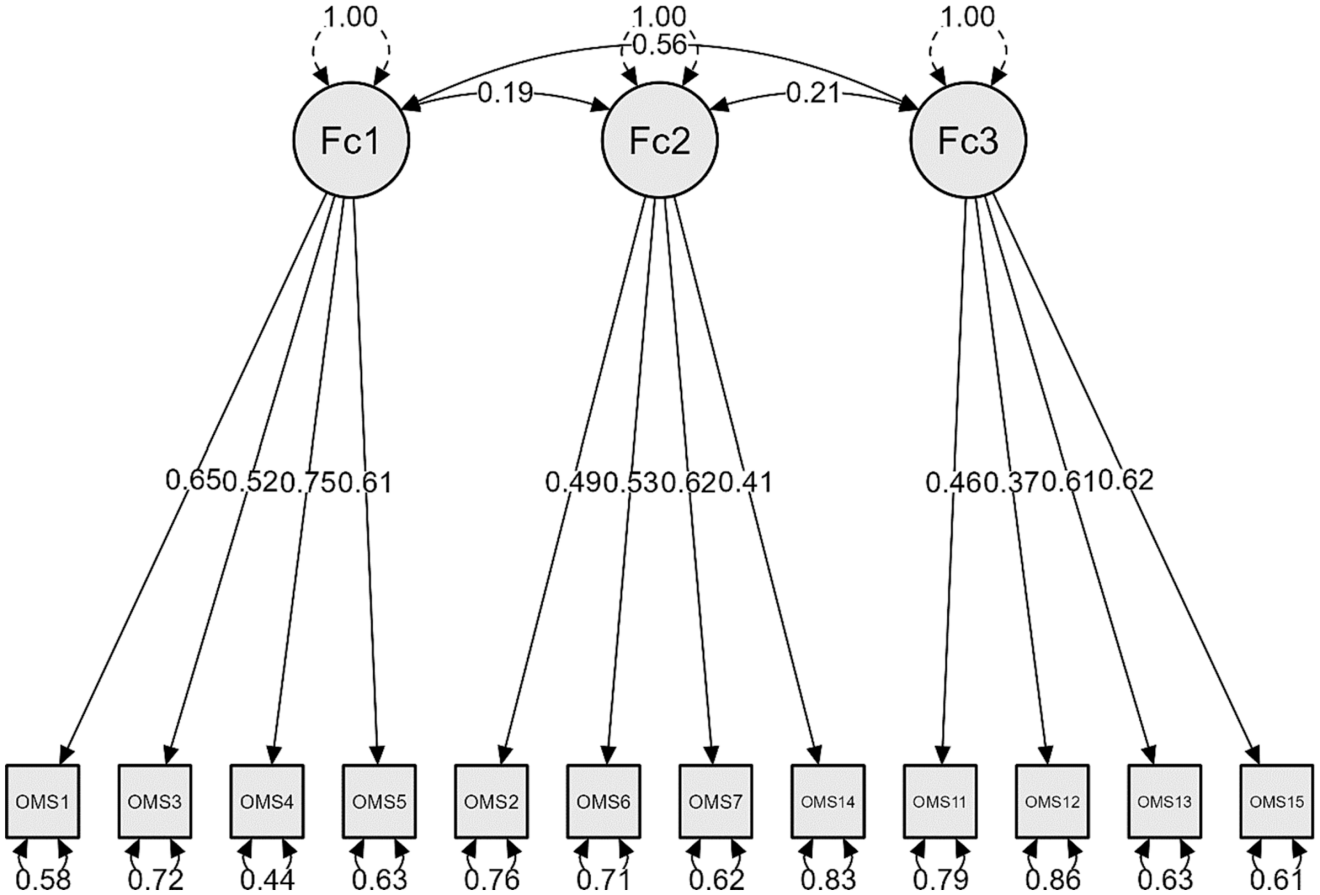
Confirmatory factor analysis of the 12-item OMS-HC scale version. *N* = 253.

Finally, we tested the 12-item structure obtained in the EFA by removing items 8, 9 and 10. This model showed good results in adjustment index values with a chi-square value of 57.969 (df = 51) and a *p* value of 0.234, CFI and TLI were above 0.95, RMSEA was below 0.06 and SRMR was below 0.08, as presented in Table 2. There was no need to correlate residual errors of items. Also, the AIC and ECVI values were lower in this model. These results suggested that this could be the most appropriate model. Figure 2 shows the structural diagram for 12-item OMS-HC scale, with loadings and correlations.

The 12-item structure obtained in EFA and confirmed in CFA was different from the structure proposed by the original authors of the scale, but considering the content of the items, it was possible to maintain the original factor names. Thus, factor one corresponded to the original factor named “Attitudes of health care providers toward disclosure and help-seeking,” factor two corresponded to the factor “Attitudes of health care providers toward people with mental illness” and factor three corresponded to the factor “Attitudes of health care providers toward social distance.” The correlation among each of the extracted factors with the total scale was strong and significant and was moderate and significant among each other (see Table 3).

**Table 3:** Correlations of OMS-HC-12 subscales with other measures.

	OMS-HC-12 items	OMS-HC-12 Factor 1	OMS-HC-12 Factor 2	OMS-HC-12 Factor 3	EDS-20
OMS-HC-12	1	0.703**	0.664**	0.720**	0.101
OMS-HC-12 Factor 1		1	0.203**	0.291**	0.120*
OMS-HC-12 Factor 2			1	0.357**	0.046
OMS-HC-12 Factor 3				1	0.057
EDS-20					1

*N* = 503. **Correlation is significant at the *p* < 0.01. *Correlation is significant at the *p* < 0.05.

### Internal consistency

The Cronbach’s alpha for the 12-item scale was 0.71, while for each factor, it was 0.67 (factor one), 0.62 (factor two) and 0.60 (factor three), respectively. The McDonald’s ω was 0.72 for the total scale, and 0.69 for factor one, 0.64 for factor two and 0.61 for factor three. The correlations among the items varied from very weak to moderate, with the maximum correlation being 0.496 and the minimum being 0.004. All items were significantly correlated with the total scale, with the minimum being 0.356 and the maximum being 0.607.

### Test–retest reliability

The intraclass correlation coefficient (ICC) was 0.65 which indicate a moderate consistency. Also, there was a significant correlation between the two moments of the scale application (*r* = 0.439*) and the results of the paired samples t-test were not significant (*p* > 0.05).

### Social desirability bias testing

Correlations between the 12-item model and the EDS-20 scale were measured, revealing no significant correlation (Pearson’s correlation 0.101, *p* = 0.080). However, factor one had a low significant correlation with the EDS-20 (Pearson’s correlation 0.120, *p* = 0.038).

### OMS-HC total and subscale scores

The 12-item OMS-HC total possible scores range from 12 to 60 with lower scores indicating less stigmatizing attitude. For the subscale scores, these can range from 4 to 20. The mean score for the 12-item OMS-HC for the total sample of this study (*N* = 503) was 39.12 (SD = 4.81), and the subscale scores for factor one were 15.37 (SD = 3.23), for factor two were 7.95 (SD = 2.59) and for factor three were 16.81 (SD = 2.51).

## Discussion

The results regarding the reliability and validity of this scale suggested that the 12-item model was the most appropriate. This version of the OMS-HC also showed an acceptable internal consistency as the original version (Modgill et al., 2014). Despite this, our results for the internal consistency of the extracted factors were lower than expected, with a Cronbach’s Alpha ranging from 0.60 to 0.67, as well as McDonald’s Omega ranging from 0.61 to 0.69. In the validation results of the original validation, Cronbach’s alpha values of less than 0.70 were also obtained (Modgill et al., 2014). In scale validation studies, it was also common for factors to have lower Cronbach’s alpha values, such as the studies conducted in Singapore (Chang et al., 2017), Hungary (Őri et al., 2020), Germany (Zuaboni et al., 2021).

The study performed in Italy (Destrebecq et al., 2018) obtained good psychometric indicators for factor structures with only two factors. Also, a recent study conducted with data from 32 European countries (Őri et al., 2023) concluded that the bifactorial structure would be the most appropriate for this scale, as suggested in the study conducted in Hungary (Őri et al., 2020). The authors (Őri et al., 2023) recommended that instead of using subscales, stigma would be assessed through the score obtained on the total scale. These results may also contribute to understanding the low values of internal consistency in the three factors extracted in our and other studies.

Our results also indicated that it would be necessary to remove the items 8, 9 and 10. Item 8 “If I had a mental illness, I would tell my friends,” appears to relate more to the shame one might experience upon receiving a mental illness diagnosis, a topic also addressed in items 3 and 4. Item 9 “Despite my professional beliefs, I have negative reactions toward people who have mental illness” mainly concerns the self-awareness of the disconnection between stigmatizing thoughts and actions. As the scale may promote self-awareness, this item might be influenced by any individual achievements while answering the questionnaire. Lastly, item 10 “There is little I can do to help people with mental illness” seems to indicate feelings of helplessness toward individuals with mental illness, which might be related to an unreasonable judgment of character, a topic also addressed in items 6, 7, 11, 12 and 14. Upon reflection, we concluded that the overall meaning of the scale was not significantly modified by removing these items. The remaining items in our scale are sufficient for assessing all domains of stigma, including labeling, stereotyping, separation, status loss and discrimination (Link and Phelan, 2001).

Validation studies in other countries also had to remove items, such as the Hungarian validation, which excluded item 14 (Őri et al., 2020), the Italian validation, which removed items 3, 8 and 9 (Destrebecq et al., 2018) and the Singaporean validation, which also removed one item (Chang et al., 2017). This can suggest that certain items may be more or less suitable depending on the cultural context in which the scale is used. Thus, our model exhibited slight differences in the subscales when compared to the original scale (Modgill et al., 2014). However, the differences were minimal and we opted to maintain the original designation of the subscales, as we considered that they were still capable of describing the constructs represented with precision.

When we assessed the correlation between OMS-HC-12 (total scale and respective subscales) and the Social Desirability Scale, we found a statistically significant correlation with the factor one “Attitudes of health care providers toward disclosure and help-seeking.” These results seem to indicate that the answers given by the participants in this study regarding disclosing a mental illness and seeking help might be based on what they thought was correct and not on their real opinion. These results may be due to the presence of self-stigma that is often present in mental illness conditions (Dubreucq et al., 2021). Nevertheless, this topic requires further study in the population of healthcare professionals.

The OMS-HC scale can serve as a tool to enhance individual reflection on stigma. Many people may not be aware that they have stigmatizing attitudes in their daily lives, but by reflecting on the questions in the scale, they may realize their preconceived notions about their stigma. Although education may change how people think about mental illness, it may not necessarily change how they behave toward individuals with it (Petkari, 2017). It is common to see people criticize others for claiming to be uncomfortable around those with mental illness, while holding same stigmas themselves. This can indicate superficiality of education on this topic. It is not unusual for individuals to take a particular stance on a subject and then fail to act accordingly (Hagerty et al., 2005). Answering this scale can help people realize that they need to deepen their understanding of mental health and consequently the stigma that may exist (Munisami et al., 2021). With the Portuguese validation of this scale, it will be possible to conduct future research into this reality in Portugal and compare different groups of healthcare professionals.

One limitation of our study relates to the sampling method, which means that the sample may not be representative of the target population. This is a commonly cited limitation of the snowball sampling data collection method. This is evident when we consider the difference between the percentage of professional groups in our sample. For this reason, the external validity is limited. Despite this limitation, our psychometric results suggest that the scale may be suitable for use with the target population.

## Conclusion

The 12-item version of the scale showed good psychometric characteristics in terms of reliability and validity. Therefore, it can be concluded that this version is appropriate to be used with the Portuguese healthcare professional population. This scale will be important for future studies to explore the prevalence of stigma and to compare stigma in different professional groups, according to professional specialty, age, and gender. In addition, it would be pertinent at the end of completing the scale to enquire the participants about the reflective potential that this scale had in terms of their individual beliefs on the subject.

## Data availability statement

The raw data supporting the conclusions of this article will be made available by the authors, without undue reservation.

## Ethics statement

The studies involving humans were approved by Comissão de Ética Centro Hospitalar S. João/ Faculdade de Medicina da Universidade do Porto under number 07/2023. The studies were conducted in accordance with the local legislation and institutional requirements. The participants provided their written informed consent to participate in this study.

## Author contributions

MBPM: Conceptualization, Data curation, Formal analysis, Investigation, Methodology, Writing – original draft, Writing – review & editing. HPP: Conceptualization, Formal analysis, Investigation, Methodology, Supervision, Validation, Visualization, Writing – review & editing. INT: Conceptualization, Data curation, Investigation, Validation, Visualization, Writing – review & editing. SM: Supervision, Validation, Visualization, Writing – review & editing. MR: Conceptualization, Data curation, Investigation, Supervision, Validation, Visualization, Writing – review & editing.
